# Online shopping self-efficacy and emotional closeness to shopping-circle members as dual pathways: the association between product familiarity and Generation Z consumers’ willingness to disclose

**DOI:** 10.3389/fpsyg.2026.1851651

**Published:** 2026-09-09

**Authors:** Mi Shi, Lanxi Wang, Miao Guo, Mengxian Chen

**Affiliations:** School of Economics and Management, Tiangong University, Tianjin, China

**Keywords:** emotional closeness to shopping-circle members, online shopping self-efficacy, product familiarity, sense of community in shopping circles, willingness to disclose

## Abstract

**Background:**

As digital natives, Generation Z exhibits distinct attitudes, values, and information behaviors, characterized by a heightened willingness to disclose personal information. This study investigates the associations between product familiarity and willingness to disclose among Generation Z consumers, with online shopping self-efficacy and emotional closeness to shopping-circle members as mediating variables and sense of community in shopping circles (particularly its emotional resonance dimension) as a moderating variable. By integrating the theory of planned behavior and social identity theory, with insights from the recently proposed vulnerability and emotional bond theory, this study develops a dual-mediation model to elucidate the psychological mechanisms underlying Generation Z’s willingness to disclose in digital consumer contexts.

**Methods:**

To test the proposed relationships, a quantitative survey was administered to 524 participants. The theoretical framework—encompassing hypothesized paths, mediating effects, and moderating effects—was examined using covariance-based structural equation modeling (CB-SEM) implemented in R.

**Results:**

Product familiarity showed a significant positive association with willingness to disclose. This association was mediated through the dual paths of online shopping self-efficacy and emotional closeness to shopping-circle members. Sense of community in shopping circles negatively moderated the relationships between product familiarity and both mediators, as well as the indirect effects of the two mediating paths.

**Conclusion:**

These findings indicate dual pathways of online shopping self-efficacy and emotional closeness to shopping-circle members linking product familiarity to willingness to disclose and highlight the boundary role of sense of community in shopping circles. The study contributes to psychological theories of information disclosure in digital environments and offers empirical insights into youth behavioral decision-making in online communities.

## 1 Introduction

Information has become a critical bridge connecting brands and consumers, exerting a profound impact on consumption decision-making processes (Heo and Lee, 2025). With the continuous evolution of the digital consumption environment, consumers’ active and passive information-sharing behaviors have become increasingly prevalent (Hu and Ou, 2025; Cui et al., 2024). Among these behaviors, self-disclosure, a key form of proactive personal information sharing, has attracted growing attention in both consumer behavior research and practical applications (Sun et al., 2026). This is particularly true for Generation Z (Tseng et al., 2025), a unique consumer group born between 1997 and 2012 (Dimock, 2019). As digital natives, Generation Z exhibits unique attitudes, values (Lyngdoh et al., 2023), and information behavior patterns that distinguish them from other generations (McKee et al., 2024). Accounting for approximately one-third of the global population, Generation Z has become a key driver of consumption growth, and its consumption influence and information behavior patterns are rapidly reshaping the global digital consumption landscape (Mason et al., 2022).

Understanding Generation Z’s self-disclosure behavior is of both theoretical and practical significance. Theoretically, it extends existing privacy and disclosure frameworks to a digital-native cohort whose norms differ fundamentally from those of previous generations. Practically, brands increasingly rely on consumer data to personalize services and cultivate loyalty, excessive disclosure requests may trigger privacy concerns and resistance. Drawing on two potential mechanisms underlying disclosure willingness—namely, online shopping problem-solving self-efficacy and emotional closeness to shopping-circle members—this study offers empirical insights for designing consumer-centric data collection strategies that effectively balance engagement with trust.

Recent studies have examined the antecedents of self-disclosure from the perspectives of privacy concerns (Bélanger et al., 2021), content intimacy (Feng et al., 2024), and cultural differences (Schumacher et al., 2023) within the context of digital consumption. However, most studies tend to treat consumers as a homogeneous group, paying insufficient attention to the self-disclosure decisions of Generation Z. Generation Z is characterized by distinct circle-based consumption attributes (Lin, 2025), and its behavioral logic across stages such as product awareness, evaluation, and decision-making differs significantly from that of other generations (El-Shihy and Awaad, 2025; Jeong and You, 2026). Compared with their counterparts in individualistic cultures, Generation Z consumers raised in collectivist cultural contexts tend to exhibit a higher willingness to disclose personal information (Wuttaphan, 2023). For this cohort, self-disclosure not only influences their purchase decisions (Kim and Jeong, 2025) but also serves as a core mechanism for brand interaction (Jeong and You, 2026). Therefore, investigating the mechanisms underlying Generation Z’s willingness to disclose in the Chinese collectivist context is essential for advancing theoretical frameworks in consumer psychology and offers an empirical basis for understanding privacy-related decision-making in the digital age.

In the digital consumption field, Generation Z’s depth of product cognition is closely associated with its self-disclosure attitudes (Rózsa et al., 2024). Product familiarity, as an important construct for measuring consumers’ level of product cognition—defined as product-level familiarity rather than brand awareness (Alba and Hutchinson, 1987)—serves as a crucial cognitive variable that bridges enterprises and consumers (Zhang Y. et al., 2021). It not only influences consumers’ information search and processing capabilities but also relates to trust formation and behavioral decision-making (Brucks, 1985; Yin et al., 2026). Although existing research has revealed the relationship between product familiarity on consumer behavior, few studies have situated product familiarity within the unique circle culture of Generation Z. Sense of community in shopping circles—particularly its emotional resonance dimension—is a critical variable rooted in Generation Z’s collectivist cultural context. It may influence circle members’ cognitive judgments and behavioral choices through the internalization of group norms and the reinforcement of social interactions (cf. Dong et al., 2025; Nguyen and Nguyen, 2025). Currently, there is a lack of systematic empirical research on whether and how the sense of community in shopping circles moderates the relationship between product familiarity and Generation Z’s willingness to disclose personal information. To fill this research gap, we focus on two constructs: online shopping self-efficacy and emotional closeness to shopping-circle members. Online shopping self-efficacy refers to consumers’ confidence in their ability to solve practical problems and manage transaction-related tasks during online shopping (cf. Bandura, 1977; see also Keith et al., 2015; Bartol et al., 2023, for a related discussion of self-efficacy in e-commerce contexts). Emotional closeness to shopping-circle members refers to the perceived emotional bonding and interpersonal connectedness that consumers develop with other members in their online shopping circles (Baumeister and Leary, 1995; Wenzel and Benkenstein, 2019). These two constructs are conceptually distinct—one capability-based and one affect-based—and both are theoretically relevant to understanding how product familiarity may relate to Generation Z’s willingness to disclose personal information.

In light of this, the present study focuses on the relationship between product familiarity and Generation Z’s willingness to disclose, with sense of community in shopping circles as a moderating variable. Through theoretical analysis and hypothesis development, this study employs path analysis and moderated effect testing using R to examine the interactive patterns, underlying mechanisms, and potential pathways through which product familiarity, sense of community in shopping circles, and related boundary factors covary with or relate to Generation Z’s willingness to disclose. Furthermore, based on the behavioral characteristics of Generation Z consumers, this study proposes targeted strategies to provide theoretical support and practical insights for consumer privacy protection and precision marketing in the digital economy, thereby contributing to the establishment of a healthy and orderly digital consumption environment.

## 2 Literature review

### 2.1 Research on Generation Z’s willingness to disclose

Willingness to disclose refers to an individual’s behavioral tendency to reveal personal information, thoughts, and feelings to others in a specific context (Kim and Dindia, 2011). It is also a crucial variable for predicting consumers’ privacy decisions and their acceptance of personalized services (Acquisti et al., 2015). With the integration of artificial intelligence and the digital economy, research perspectives on consumer self-disclosure have expanded from interpersonal communication to human-computer interaction (Pelau et al., 2025) and brand-consumer relationships (Wang, 2025).

As digital natives, Generation Z’s information-sharing behavior on social media is influenced by unique psychological mechanisms and exhibits contradictory characteristics, being highly sensitive to privacy risks while simultaneously displaying a strong willingness to disclose personal information for personalized services and social recognition (Rózsa et al., 2024). Existing research on factors influencing Generation Z’s willingness to disclose has examined individual traits (e.g., self-esteem, Wuttaphan, 2023), interpersonal relationships (Kim and Jeong, 2025), and situational factors like platform characteristics (El-Shihy and Awaad, 2025). However, despite product familiarity being a key cognitive variable that bridges enterprises and consumers, existing research has rarely incorporated it into the analytical framework of Generation Z’s willingness to disclose.

### 2.2 Product familiarity, sense of community in shopping circles, and Generation Z’s willingness to disclose

Online interest-based circles, formed around shared consumption preferences, have become a crucial social context where Generation Z acquires product information and constructs consumption meaning (Lin, 2025). Product familiarity denotes the consumer’s accumulated number of product-related experiences (Alba and Hutchinson, 1987). As a key variable at the individual cognitive level (Brucks, 1985; Zhang Y. et al., 2021), Product familiarity along with sense of community, a core construct at the psychosocial level (Dong et al., 2025), which constitutes important variables for describing Generation Z’s willingness to disclose.

Online interest-based circles, formed around shared consumption preferences, have become a crucial social context where Generation Z acquires product information and constructs consumption meaning (Lin, 2025). Product familiarity denotes the consumer’s accumulated number of product-related experiences (Alba and Hutchinson, 1987). As a key variable at the individual cognitive level (Brucks, 1985; Zhang Y. et al., 2021), product familiarity, along with sense of community in shopping circles—particularly its emotional resonance dimension—a core construct at the psychosocial level (Dong et al., 2025), constitutes important variables for describing Generation Z’s willingness to disclose.

Existing research indicates that product cues are closely associated with information disclosure behavior. As a key representation of consumers’ perception of product reliability, product familiarity forms a relevant basis for understanding how consumers construct their identity through circle interactions (Yin et al., 2026). The early enrichment hypothesis suggests that product familiarity may be associated with consumers’ ability to acquire new information (Chase and Simon, 1973), which is consistent with the possibility that individuals with higher familiarity are may be more likely to capture information cues within circles. Moreover, consumers with high familiarity tend to align with the mainstream views of their circle, which corresponds to a stronger sense of belonging and a higher reported self-disclose intention (Zhang C. et al., 2021).

Unlike the individual cognitive dimension represented by product familiarity, sense of community in shopping circles is rooted in the emotional connection between an individual and the community (Bang and Han, 2025). Sense of virtual community is defined as “members’ feelings of belonging, attachment, identity, and mutual influence in a technology-supported group” (Blanchard and Markus, 2004). This concept is further operationalized into three dimensions—membership belongingness, influence, and immersion (Koh and Kim, 2003). This implies that when Generation Z consumers develop strong emotional identification with a particular circle, their information processing may be related to the sense of community. In other words, sense of community in shopping circles may moderate how product familiarity is associated with willingness to disclose.

Sense of community in shopping circles—specifically its emotional resonance dimension—refers to the perceived emotional similarity and affective resonance that individuals experience with other members of their shopping circle, rooted in the emotional bonding dimension of social identity theory (Tajfel and Turner, 1986) and drawing from the broader literature on sense of community (McMillan and Chavis, 1986). In this study, we adopt this emotional resonance facet of sense of community rather than its full multidimensional conceptualization (belonging, identification, influence, and membership), as our measure is specifically designed to capture the affective similarity that individuals perceive with other circle members.

Accordingly, this study integrates insights from the theory of planned behavior and the recently proposed vulnerability and emotional bond theory (VTEB; Epstein et al., 2025), introducing online shopping self-efficacy and emotional closeness to shopping-circle members as mediating variables to systematically examine potential mechanisms that may account for the association between product familiarity and Generation Z’s willingness to disclose.

## 3 Research hypotheses

Thus, product familiarity not only improves judgment of information value but also strengthens confidence in effective sharing.

According to social cognitive theory, self-efficacy is shaped by direct experiences, vicarious experiences, and social persuasion (Bandura, 1977). Within circle interactions, product familiarity is positively associated with online shopping self-efficacy through two potential pathways. On the one hand, it sharpens individuals’ perception of the value of their product-related knowledge (Polman et al., 2022). Familiar consumers possess a deeper understanding of product attributes and usage, enabling them to judge what information is valuable to circle members (Tang and Marinova, 2020). On the other hand, product familiarity confers greater discursive authority (Fang et al., 2019; Cheung et al., 2014), reducing concerns about being questioned and reinforcing confidence through positive feedback (cf. Bartol et al., 2023). Thus, product familiarity not only improves judgment of information value but also strengthens confidence in effective sharing within online shopping circles.

Such optimistic expectations are associated with lower psychological resistance to disclosure (Keith et al., 2015). Additionally, high self-efficacy individuals may be more motivated to consolidate their status by demonstrating expertise, which corresponds to higher disclosure intention. In other words, consumers who believe they can effectively manage their personal information perceive lower privacy risks and are therefore more willing to disclose.

The theory of planned behavior posits that self-efficacy, as perceived behavioral control, is a core antecedent of behavioral intention (Ajzen, 1991, 2002). In online shopping circle contexts, individuals with high online shopping self-efficacy are more likely to believe their information is valuable and will generate positive outcomes (Shneor and Munim, 2019). Such optimistic expectations are associated with lower psychological resistance to disclosure (cf. Bartol et al., 2023). Additionally, high self-efficacy individuals may be more motivated to consolidate their status by demonstrating expertise, which corresponds to higher disclosure intention. In other words, consumers who feel confident in navigating online shopping environments and managing transaction-related tasks perceive lower privacy risks and are therefore more willing to disclose.

Taken together, product familiarity is positively associated with online shopping self-efficacy, and online shopping self-efficacy is, in turn, positively associated with willingness to disclose. Accordingly, online shopping self-efficacy is proposed as a potential mediating mechanism in the relationship between product familiarity and disclosure intention.

Based on the above reasoning, we propose the following hypotheses:

*H1a*: Product familiarity is positively associated with online shopping self-efficacy.

*H1b*: Online shopping self-efficacy is positively associated with willingness to disclose.

*H1*: Online shopping self-efficacy mediates the relationship between product familiarity and willingness to disclose.

Emotional closeness to shopping-circle members, a key dimension of tie strength and a robust indicator of relational intensity, refers to the degree of emotional bonding formed through frequent interaction and mutual understanding with other members in the shopping circle, characterized by trust, care, and reliance (Granovetter, 1973). It is further conceptualized within the vulnerability theory of emotional bonding (VTEB) as “a bond formed between two individuals through shared intense emotions, understanding, and empathy,” aligning with the interpersonal process model (Epstein et al., 2025). In the context of shopping circles, emotional closeness to shopping-circle members may thus serve as a potential bridge between product familiarity and willingness to disclose.

Product familiarity may be positively associated with emotional closeness to shopping-circle members. Circle members often gather around shared consumption interests, and common product knowledge forms the cognitive basis for interaction. Individuals with high product familiarity may be better able to assess information value, producing content that garners recognition from others (Shamayleh and Arsel, 2026). Shared product cognition may also contribute to psychological compatibility, and the perception of “like-mindedness” may correspond to reduced emotional distance. Familiar product topics may further be associated with greater interaction frequency and depth, potentially enabling communication to extend from superficial exchange to emotional resonance (Wang et al., 2025). Thus, product familiarity shows a positive relationship with emotional closeness to shopping-circle members, reflected in improved interaction quality and reinforced psychological compatibility.

Emotional closeness to shopping-circle members may be positively associated with willingness to disclose. According to Epstein et al. (2025), strong emotional closeness creates a psychologically safe atmosphere, which may make individuals more likely to believe their disclosure will receive support from circle members (Laurenceau et al., 1998). Su et al. (2025) found that users interacting with strong emotional bonds are more inclined to share personalized content, driven by a “desire to be liked.” Additionally, motivated to maintain and deepen bonds, individuals may proactively disclose information to consolidate relationships (Wang, 2024). Hence, strong emotional closeness to shopping-circle members not only provides a safe context for disclosure but also corresponds to an intrinsic motivation for it.

Integrating the above analysis, we propose the following hypotheses:

*H2a*: Product familiarity is positively associated with emotional closeness to shopping-circle members.

*H2b*: Emotional closeness to shopping-circle members is positively associated with willingness to disclose personal information.

*H2*: Emotional closeness to shopping-circle members mediates the relationship between product familiarity and willingness to disclose personal information.

According to the Elaboration Likelihood Model (ELM; Petty and Cacioppo, 1986), individuals process information via either a central or peripheral route depending on their involvement. Sense of community in shopping circles—particularly its emotional resonance component—may be viewed as a situational involvement factor. Individuals with higher emotional resonance with their circle tend to engage in deeper, central-route processing, and may be more likely to treat the circle as part of their self-extension (Belk, 2016; Honarvar and Sepehrinia, 2025). This may in turn be associated with their ability to derive value beliefs from product familiarity, which may correspond to higher online shopping self-efficacy. In contrast, individuals with lower emotional resonance may process information more shallowly via the peripheral route, which may make it less likely for them to form the efficacy belief that “my information is valuable,” even when they are familiar with the product (Trzebiński and Marciniak, 2022).

Based on this reasoning, we propose the following hypothesis:

*H3*: The positive relationship between product familiarity and online shopping self-efficacy is stronger at higher levels of sense of community (emotional resonance dimension) than at lower levels.

Social identity theory suggests that excessive group identification may foster homogeneous information environments and normative pressures (Turner et al., 1987). In shopping circles, highly identified members tend to conform or remain silent due to normative pressure (Algesheimer et al., 2005), while dynamic social norms shape individual opinions through informational cascades (Feng et al., 2021). As a result, individuals with high product familiarity but views that deviate from the mainstream may suppress authentic emotional expression for fear of being questioned or excluded, forming a group-based self-censorship. Meanwhile, strong sense of community (emotional resonance dimension) could lead individuals to prioritize interacting with “their own kind”, thereby potentially inducing selective closure and limiting diversified exchanges (Coleman, 1988). Self-censorship and selective closure together constitute an inhibitory mechanism that may attenuate the positive effect of product familiarity on emotional closeness to shopping-circle members. This logic is also consistent with the behavioral characteristics of individuals with an interdependent self-construal (i.e., a “we”- orientation), who prioritize group harmony and avoid relational risks (Zhang C. et al., 2021; Zhang Y. et al., 2021).

Accordingly, we propose H4: The positive relationship between product familiarity and emotional closeness to shopping-circle members is weaker when individuals perceive higher sense of community (emotional resonance dimension) than when they perceive lower sense of community.

Consistent with social identity theory, when the sense of community in shopping circles (emotional resonance dimension) is high, individuals tend to internalize circle values into their self-concept and perceive their information as valuable to the circle (Ashforth and Mael, 1989). This identification may be associated with a stronger positive relationship between product familiarity and online shopping self-efficacy (Polman et al., 2022), thereby potentially enhancing the role of self-efficacy in shaping information disclosure. Conversely, high sense of community (emotional resonance dimension) may also coincide with homogenization pressure and selective closure, which could weaken the transformation of product familiarity into emotional closeness to shopping-circle members, potentially reducing the pathway through emotional closeness (Baumeister and Leary, 1995). This pattern aligns with prior findings that a salient collective orientation (i.e., “we”) may suppress disclosure by undermining the development of emotional closeness (Zhang C. et al., 2021; Zhang Y. et al., 2021). Thus, under high emotional resonance, the two mediating pathways may respond differently. Although competing logics exist (e.g., group norms may override individual cognition), we draw primarily on the logic that high sense of community (emotional resonance dimension) enhances individuals’ perception that their information is valuable to the circle, leading us to predict a stronger self-efficacy pathway. Conversely, homogenization pressure suggests a weaker emotional closeness pathway.

When sense of community in shopping circles (emotional resonance dimension) is low, individuals lack a strong sense of belonging, making it difficult to form the belief that their information is valuable to the circle. Consequently, the positive relationship between product familiarity and online shopping self-efficacy tends to be weaker, and the mediating role of online shopping self-efficacy in disclosure is correspondingly reduced (Chiu et al., 2006). Likewise, product familiarity is less likely to be positively associated with deep emotional bonds, and the mediating role of emotional closeness to shopping-circle members is also weaker.

Based on the above reasoning, the following hypotheses are proposed.

*Hypothesis H5*: The indirect pathway from product familiarity to willingness to disclose via online shopping self-efficacy varies as a function of sense of community (emotional resonance dimension), being stronger when sense of community is higher.

*Hypothesis H6*: The indirect pathway from product familiarity to willingness to disclose via emotional closeness to shopping-circle members varies as a function of sense of community (emotional resonance dimension), being weaker when sense of community is higher.

To minimize potential demographic biases, this study includes gender, education level, and income as control variables (Rózsa et al., 2024). These controls help reduce confounding bias and improve the precision of estimated relationships, though causal claims are not warranted given the single-occasion correlational design.

**Figure 1:**
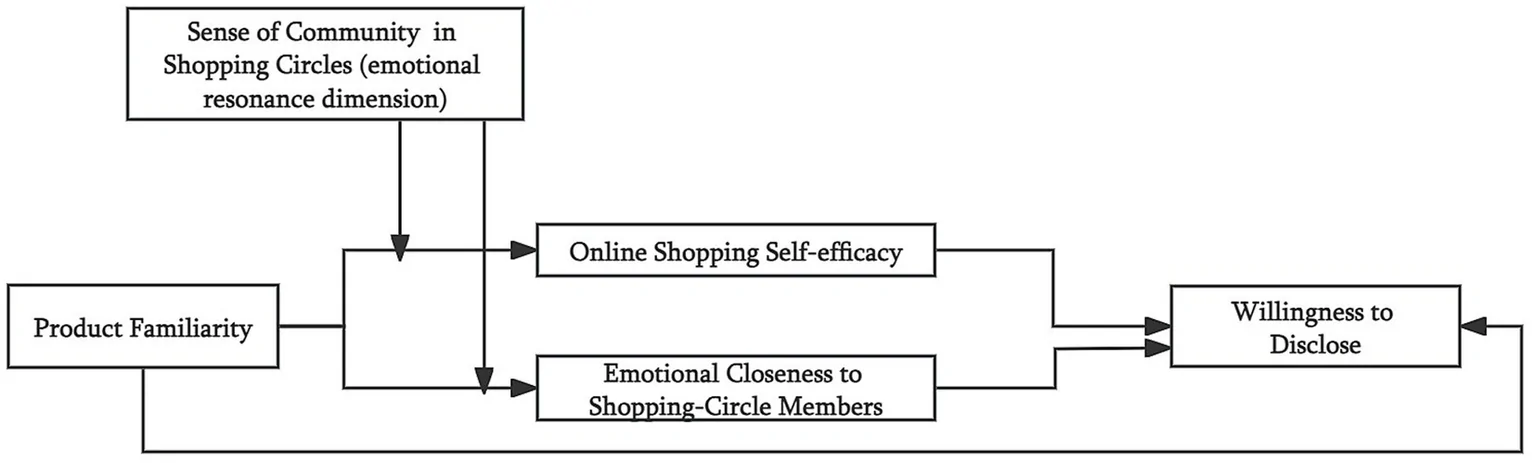
Theoretical model.

Figure 1 presents the theoretical framework. The model integrates the theory of planned behavior, social identity theory, and the emerging vulnerability theory of emotional bonding (VTEB) to propose a conceptual framework for how product familiarity, in interaction with sense of community (emotional resonance with the shopping circle), may be statistically associated with Generation Z’s willingness to disclose, with online shopping self-efficacy and emotional closeness to shopping-circle members as statistical mediators.

## 4 Research design

### 4.1 Research sample

This study surveyed Generation Z consumers who shop on various e-commerce platforms in China, such as Douyin (TikTok), Dewu, Meituan, Pinduoduo, and WeChat shopping groups. The survey platform^1^ was programmed with a pre-screening filter that restricted participation to individuals born between 1997 and 2009 (i.e., Generation Z, consistent with Dimock, 2019), ensuring that all respondents were aged 18 years or older at the time of the survey. Data were collected between April 29 and May 30, 2022, through the professional online survey platform https://www.Wenjuanxing.com using targeted distribution. A total of 545 questionnaires were initially collected. After systematic screening, questionnaires with response times below 30 s or above 900 s were manually excluded, resulting in 524 valid responses. The validity of the questionnaires was further ensured through a combination of systematic screening and manual review. All respondents had prior experience with online shopping and engagement in online social circles, which supports the representativeness of the sample. During the survey, the research team explicitly informed respondents that the collected data would be used for academic research purposes only and pledged strict confidentiality. Minors under the age of 18 were excluded from the sample, as their online activities—including independent online shopping—are subject to legal restrictions under China’s laws on the protection of minors. To ensure the quality of measurement instruments and sample representativeness, this study adopted a two-independent cross-sectional sample design for data collection, with no overlap between respondents. This design draws on the methodological approach of van Dijk et al. (2022).

To assess whether the pretest and formal test samples could be combined, multi-group confirmatory factor analysis (MG-CFA) with MLR estimation was conducted across the two independent subsamples (n₁ = 219 for the pretest; n₂ = 305 for the formal test).

As shown in Table 1, configural invariance was supported by acceptable model fit (CFI = 0.934, RMSEA = 0.064). Metric invariance was established (ΔCFI = −0.003, ΔRMSEA = 0.000), and scalar invariance was further supported (ΔCFI = −0.007, ΔRMSEA = 0.002), with all ΔCFI values above the recommended cutoff of −0.01 (Cheung and Rensvold, 2002). These results demonstrate that respondents in both subsamples interpreted the measurement items consistently, with equivalent factor loadings and intercepts across samples. Given the established invariance across the two subsamples, the MG-CFA results provide empirical support for combining the two samples for the main structural analyses. The final combined sample (N = 524) provides more stable and reliable parameter estimates for the subsequent structural equation modeling analyses than either sample alone (van Dijk et al., 2022). This two independent cross-sectional sample design follows established methodological recommendations for instrument development and validation (Nunnally, 1978), and the absence of post-pretest modifications further justifies sample combination without inflating Type I error.

**Table 1:** MG CFA across the two independent subsamples (N_1_ = 219, N_2_ = 305).

Model	*df*	*χ* ^2^	CFI	RMSEA (90% CI)	ΔCFI	ΔRMSEA
Configural invariance	578	1197.095	0.934	0.064 [0.059, 0.069]	—	—
Metric invariance	599	1247.694	0.931	0.064 [0.059, 0.070]	−0.003	0.000
Scalar invariance	620	1335.357	0.924	0.067 [0.062, 0.071]	−0.007	0.002

The detailed demographic characteristics of the combined sample (*N* = 524) are presented in Table 2. The gender distribution was balanced, with 49.43% male and 50.57% female participants. In terms of educational attainment, 66.22% of the sample held a bachelor’s degree, 29.01% held a master’s degree, 4.01% held a doctoral degree or higher, and 0.76% had less than a bachelor’s degree. Regarding monthly income, 39.12% of respondents reported a monthly disposable income of 5,000 RMB or less, 41.03% reported between 5,001 and 10,000 RMB, and 19.85% reported 10,001 RMB or more. The sample broadly reflects the demographic characteristics of Chinese Generation Z online consumers reported in previous studies.

**Table 2:** Demographic characteristics of respondents (*N* = 524).

Characteristics	Frequency	Percentage
Gender		
Male	259	49.43%
Female	265	50.57%
Educational attainment		
Less than bachelor’s degree	4	0.76%
Bachelor’s degree	347	66.22%
Master’s degree	152	29.01%
Doctoral degree or higher	21	4.01%
Monthly income		
5,000 RMB or less	205	39.12%
5,000 to 10,000 RMB	215	41.03%
10,000 RMB or more	104	19.85%

### 4.2 Variable measurement

This study employs five constructs, namely product familiarity, sense of community in shopping circles, online shopping self-efficacy, emotional closeness to shopping-circle members, and willingness to disclose (see Appendix A for all measurement items).

Four of these scales were adapted from well-validated mature scales developed in both domestic and international literature and were adjusted to fit the consumption context of Generation Z in China. The English versions of these scales were first translated into Chinese independently by three bilingual researchers. After reaching a consensus on the translated versions, the Chinese versions were then back-translated into English by two independent translators to ensure semantic equivalence with the original scales. Minor wording adjustments were made to enhance clarity and contextual relevance for the Chinese Generation Z sample. To ensure the alignment between the theoretical constructs and the measurement items, we conducted a content validity assessment prior to data collection. Four experts in consumer behavior and scale development were invited to evaluate the relevance, representativeness, and clarity of each item relative to its intended construct. Based on their feedback, minor revisions were made to the wording of several items to improve conceptual precision and contextual fit. The content validity index (CVI) at the item level (I-CVI) ranged from 0.85 to 1.00, and the scale-level CVI (S-CVI) exceeded 0.90, indicating strong content validity. These procedures ensure that the measurement items adequately capture the intended constructs and that the item–construct alignment is empirically supported.

To further clarify the scale development process, the pretest subsample served solely to verify, not refine, the factor structure of the newly developed sense of community in shopping circles scale. No items were added, deleted, or modified based on the pretest results, and no hypothesis testing regarding the structural model was conducted on the pretest data. Consequently, there was no risk of data peeking or p-hacking.

The sense of community in shopping circles scale was developed specifically for the context of this study. In this study, we focus specifically on the emotional resonance facet of sense of community, rather than its broader multidimensional conceptualization (belonging, identification, influence, and membership), as our measure is designed to capture the affective similarity that individuals perceive with other circle members (see Appendix A for all measurement items). All constructs were measured using a five-point Likert scale ranging from 1 (strongly disagree) to 5 (strongly agree).

Product familiarity was measured by referencing the definition and measurement approach from Zhang Y. et al. (2021). Based on this research context, we further developed a five-item scale adapted from the localized scale developed by Loureiro et al. (2020), (e.g., “I am very familiar with the functions and features of this product”), using a 5-point Likert scale. All items focused on product-level knowledge (e.g., product features, functions, usage experience). In this study, the Cronbach’s α coefficient for this scale was 0.883.

Sense of community in shopping circles was self-developed based on the conceptual definition of sense of community (McMillan and Chavis, 1986) and sense of circle in mobile social networks (Jin et al., 2018), consisting of six items. In this study, we focus specifically on the emotional resonance dimension of sense of community, rather than its full multidimensional conceptualization, as our measure is designed to capture the affective similarity that individuals perceive with other circle members. A representative item is: “I share similar emotional perceptions and response patterns with members of my online shopping circles.” In the pretest phase, this scale underwent item refinement through exploratory factor analysis, the specific results of which are shown in Table 3. In the combined sample, the scale demonstrated good internal consistency, with a Cronbach’s α value of 0.900.

**Table 3:** Exploratory factor analysis results of SCSC scale (N_1_ = 219).

Scale	Items	Factor loading	Communality (*h*^2^)	KMO	Bartlett’s test of sphericity	Variance explained
SCSC	SCSC1	0.77	0.59	0.71	*χ*^2^(15) = 618.30, *p* < 0.001	48%
	SCSC2	0.62	0.39			
	SCSC3	0.71	0.50			
	SCSC4	0.72	0.51			
	SCSC5	0.55	0.30			
	SCSC6	0.76	0.58			

PF, product familiarity; SCSC, sense of community in shopping circles (emotional resonance dimension); SE, online shopping self-efficacy; EC, emotional closeness to shopping-circle members; WD, willingness to disclose.

Online shopping self-efficacy was measured using the four-item scale developed by Liu et al. (2017). A representative item is “As long as I make an effort, I can always solve the difficult problems that arise during online shopping.” In this study, the Cronbach’s *α* coefficient for this scale was 0.877.

Emotional closeness to shopping-circle members developed a five-item scale by adapting and extending the three-item emotional closeness scale originally developed by Kong and Liang (2017) to fit the context of social commerce. A representative item is “Purchasing products on social commerce websites makes me feel close to members of my shopping group/circle.” In this study, the Cronbach’s α coefficient for this scale was 0.881.

Willingness to disclose was measured using the six-item willingness to disclose scale adapted from the self-disclosure scale developed by Malhotra et al. (2004), and augmented to suit the online shopping context in China. In this study, personal information willing to disclose refers to demographic data (e.g., age, location), contact details (e.g., email address, phone number), and consumption-related information (e.g., purchase history, product reviews). The survey context was specified as social commerce platforms (e.g., Xiaohongshu, Douyin) and product-operated online communities (e.g., WeChat shopping groups, product official apps). A representative item is: “I am willing to proactively provide my real personal information in online shopping groups or niche communities for products I have purchased.” In this study, the Cronbach’s α coefficient for this scale was 0.897.

### 4.3 Statistical analysis

All statistical analyses were conducted in R version 4.5.1 (2025-06-13) using the lavaan, psych, dplyr, haven, ggplot2, and interactions packages. EFA was conducted using principal-axis factoring with varimax rotation. CFA and the structural equation models were estimated using robust maximum likelihood (MLM), while measurement invariance was assessed using robust maximum likelihood (MLR). Mediation effects were evaluated using bias-corrected bootstrap confidence intervals based on 5,000 resamples, with MLM estimation used as a robustness check.

All measurement items were assessed on five-point Likert scales (1 = strongly disagree, 5 = strongly agree) and were treated as approximately continuous indicators in the CFA and SEM analyses. Missing observations were handled using listwise deletion (na.omit), resulting in 524 complete cases. The CFA and SEM models specified product familiarity (PF), sense of community in shopping circles (SCSC), online shopping self-efficacy (SE), emotional closeness to shopping-circle members (EC), and willingness to disclose (WD) as latent variables rather than composite scores. For moderation analyses, latent factor scores were obtained using lavPredict(), mean-centered, and multiplied to construct the PF × SCSC interaction term. Simple-slope analyses were then conducted at ±1 SD of SCSC. Descriptive statistics and Pearson correlations were calculated using construct-level mean scores. The final sample size was determined by the available eligible complete responses from the two independent samples rather than by a formal *a priori* power analysis.

## 5 Empirical analysis

### 5.1 Exploratory factor analysis

Exploratory factor analysis (EFA) was conducted to assess the factor structures of all constructs using the first-wave sample (N_1_ = 219), which was independent of the formal analysis sample. Principal axis factoring was employed as the extraction method.

Self-developed scale (SCSC). EFA was performed on the six items of the self-developed sense of community in shopping circles (SCSC) scale. The Kaiser–Meyer–Olkin (KMO) measure was 0.71, and Bartlett’s test of sphericity was significant (*χ*^2^(15) = 618.30, *p* < 0.001), verifying the suitability of the data for factor analysis. Using principal axis factoring, a single factor was extracted, accounting for 48% of the total variance. As shown in Table 3, factor loadings of all items ranged from 0.55 to 0.77, and communalities (*h*^2^) ranged from 0.30 to 0.59, demonstrating a sound unidimensional structure of the SCSC scale.

Established scales. EFA was also performed on the four established scales (product familiarity, online shopping self-efficacy, emotional closeness to shopping-circle members, and willingness to disclose) using the first subsample (N₁ = 219). Principal axis factoring with varimax rotation was applied, and one factor was extracted for each scale in line with its original theoretical structure. As shown in Table 4, KMO values for the four scales ranged from 0.67 to 0.82, and Bartlett’s tests of sphericity were all significant (*p* < 0.001), confirming the appropriateness of factor analysis for each scale. All item factor loadings exceeded the recommended threshold of 0.50 (ranging from 0.58 to 0.87), and communalities (*h*^2^) ranged from 0.33 to 0.76. The variance explained by each factor ranged from 45 to 53%. These results verified the sound unidimensionality of the established scales in the present study.

**Table 4:** Exploratory factor analysis results of established scales (N_1_ = 219).

Scale	Items	Factor loading	Communality (*h*^2^)	KMO	Bartlett’s test of sphericity	Variance explained
PF	PF1	0.77	0.60	0.79,	*χ*^2^ (10) = 481.66, *p* < 0.001	53%
	PF2	0.73	0.54			
	PF3	0.64	0.40			
	PF4	0.78	0.61			
	PF5	0.72	0.52			
SE	SE1	0.77	0.60	0.68	*χ*^2^ (6) = 342.15, *p* < 0.001	53%
	SE2	0.63	0.39			
	SE3	0.60	0.36			
	SE4	0.87	0.76			
EC	EC1	0.74	0.55	0.82	*χ*^2^ (10) = 379.63, *p* < 0.001	49%
	EC2	0.71	0.50			
	EC3	0.60	0.36			
	EC4	0.76	0.57			
	EC5	0.69	0.47			
WD	WD1	0.72	0.52	0.67	*χ*^2^ (15) = 558.13, *p* < 0.001	45%
	WD2	0.67	0.45			
	WD3	0.68	0.47			
	WD4	0.62	0.39			
	WD5	0.58	0.33			
	WD6	0.75	0.56			

### 5.2 Measurement model assessment

Confirmatory factor analysis (CFA) was conducted to evaluate the measurement model of the five constructs: product familiarity, sense of community in shopping circles, online shopping self-efficacy, emotional closeness to shopping-circle members, and willingness to disclose. As shown in Table 5, the hypothesized five-factor model demonstrated a good fit to the data (*χ*^2^/df = 2.274, RMSEA = 0.049, CFI = 0.953, TLI = 0.947, SRMR = 0.036) and outperformed all alternative models, supporting the distinctiveness of the five constructs. The CFA was performed on the combined sample (N = 524).

**Table 5:** Results of confirmatory factor analysis (*N* = 524).

Model	Factor structure	*χ* ^2^	*df*	*χ*^2^/*df*	RMSEA	CFI	TLI	SRMR
Five-factor model	PF, SCSC, SE, EC, WD	657.270	289	2.274	0.049	0.953	0.947	0.036
Four-factor model 1	PF + SE, SCSC, EC, WD	1471.413	293	5.022	0.088	0.850	0.834	0.072
Four-factor model 2	PF, SE + EC, SCSC, WD	1429.167	293	4.878	0.086	0.855	0.840	0.067
Three-factor model	PF + SE, EC + WD, SCSC	2557.924	296	8.642	0.121	0.712	0.684	0.116
Two-factor model	PF + SE + EC + WD, SCSC	3256.048	298	10.926	0.138	0.623	0.589	0.112
Single-factor model	PF + SE + EC + SCSC+WD	4224.860	299	14.130	0.158	0.500	0.457	0.131

PF, product familiarity; SCSC, sense of community in shopping circles; SE, self-efficacy; EC, emotional closeness; WD, willingness to disclose. The symbol + indicates the combination of factors.

All standardized factor loadings exceeded the recommended threshold of 0.70, as presented in Table 6, confirming adequate indicator reliability. Composite reliability and Cronbach’s α values both ranged from 0.877 to 0.900, exceeding the 0.70 benchmark and indicating strong internal consistency (Bagozzi and Yi, 1988). The average variance extracted (AVE) for each construct ranged from 0.594 to 0.641, above the 0.50 threshold, thereby supporting convergent validity (Fornell and Larcker, 1981).

**Table 6:** Reliability and convergent validity (*N* = 524).

Construct	Items	Std. loading	Cronbach’s *α*	CR	AVE
Product familiarity	PF1	0.780	0.883	0.883	0.602
	PF2	0.797			
	PF3	0.733			
	PF4	0.796			
	PF5	0.772			
Sense of community in shopping circles	SCSC1	0.776	0.900	0.900	0.600
	SCSC2	0.757			
	SCSC3	0.799			
	SCSC4	0.791			
	SCSC5	0.719			
	SCSC6	0.802			
Self-efficacy	SE1	0.809	0.877	0.877	0.641
	SE2	0.769			
	SE3	0.773			
	SE4	0.849			
Emotional closeness	EC1	0.778	0.881	0.881	0.598
	EC2	0.816			
	EC3	0.716			
	EC4	0.779			
	EC5	0.775			
Willingness to disclose	WD1	0.792	0.897	0.898	0.594
	WD2	0.778			
	WD3	0.744			
	WD4	0.763			
	WD5	0.758			
	WD6	0.788			

The Fornell–Larcker criterion and the heterotrait-monotrait (HTMT) ratio were adopted to assess discriminant validity. As shown in Table 7, the square root of the average variance extracted (AVE) for each construct (diagonal elements) exceeded its correlations with other constructs. Additionally, all HTMT values in Table 8 were below the conservative threshold of 0.85 (Henseler et al., 2015). These results collectively confirm that discriminant validity is established.

**Table 7:** Fornell–Larcker discriminant validity.

Construct	1	2	3	4	5
1. PF	0.776				
2. SCSC	0.548	0.775			
3. SE	0.457	0.377	0.801		
4. EC	0.515	0.396	0.483	0.773	
5. WD	0.353	0.395	0.431	0.402	0.771

**Table 8:** HTMT discriminant validity.

Construct	1	2	3	4	5
1. PF	1.000				
2. SCSC	0.547	1.000			
3. SE	0.459	0.379	1.000		
4. EC	0.511	0.391	0.485	1.000	
5. WD	0.356	0.393	0.431	0.402	1.000

To further validate the measurement structure, we re-estimated the five-factor measurement model using the formal subsample (N_2_ = 305) as a robustness check. As shown in Table 9, the model demonstrated excellent fit to the data: Satorra–Bentler scaled *χ*^2^(289) = 334.54, CFI = 0.991, TLI = 0.990, RMSEA = 0.023 (90% CI [0.007, 0.033]), and SRMR = 0.036. All fit indices exceeded the recommended cutoffs (Hu and Bentler, 1999), confirming the robust factor structure of the five constructs in the formal subsample. Table 10 further reports the standardized factor loadings, composite reliability (CR), and average variance extracted (AVE) for each construct. All loadings were significant (*p* < 0.001), ranging from 0.777 to 0.880. CR values ranged from 0.901 to 0.929, exceeding 0.70, and AVE values ranged from 0.647 to 0.720, all above 0.50. These results are consistent with those from the combined sample (N = 524), further confirming the stability of the measurement model.

**Table 9:** Confirmatory factor analysis results for the formal subsample (N_2_  =  305).

Fit index	Value
*χ*^2^ (Satorra‑Bentler scaled)	334.54
df	289
CFI	0.991
TLI	0.990
RMSEA	0.023
RMSEA 90% CI‑lower	0.007
RMSEA 90% CI‑upper	0.033
SRMR	0.036

CFI, comparative fit index; TLI, Tucker–Lewis index; RMSEA, root mean square error of approximation; CI, confidence interval; SRMR, standardized root mean square residual. Robust maximum likelihood (MLM) estimation was used.

**Table 10:** Standardized factor loadings, CR, and AVE for the formal subsample (N_2_ = 305).

Construct	Items	Std. loading	CR	AVE
PF	PF1	0.787	0.901	0.647
	PF2	0.827		
	PF3	0.793		
	PF4	0.815		
	PF5	0.798		
SCSC	SCSC1	0.832	0.928	0.684
	SCSC2	0.845		
	SCSC3	0.838		
	SCSC4	0.837		
	SCSC5	0.800		
	SCSC6	0.809		
SE	SE1	0.833	0.911	0.720
	SE2	0.845		
	SE3	0.870		
	SE4	0.846		
EC	EC1	0.829	0.912	0.676
	EC2	0.880		
	EC3	0.777		
	EC4	0.802		
	EC5	0.819		
WD	WD1	0.838	0.929	0.686
	WD2	0.850		
	WD3	0.780		
	WD4	0.842		
	WD5	0.844		
	WD6	0.815		

PF, product familiarity; SCSC, sense of community in shopping circles (emotional resonance dimension); SE, online shopping self-efficacy; EC, emotional closeness to shopping-circle members; WD, willingness to disclose. All factor loadings are significant at *p* < 0.001. CR, composite reliability; AVE, average variance extracted.

### 5.3 Common method bias test

The Harman’s single-factor test was conducted to assess potential common method bias in this study. An exploratory factor analysis (EFA) was performed on all measurement items across the five constructs. The results revealed that the five extracted factors accounted for a total variance of 68.44%, with the first factor explaining only 34.74% of the cumulative variance. This value falls below the recommended threshold of 40%, providing preliminary evidence that common method bias is within an acceptable range.

In addition, a common latent factor (CLF) was introduced into the original measurement model, allowing all items to load on both their respective constructs and the CLF. The comparison between the CLF-adjusted model and the original model showed negligible changes in model fit indices (ΔRMSEA = 0.000, ΔCFI = 0.004, ΔTLI = 0.002, ΔSRMR = 0.001). These results indicate that the inclusion of a common method factor did not substantially alter model fit, further confirming that common method bias does not pose a serious threat to the validity of this study (Tang and Wen, 2020).

### 5.4 Descriptive statistics and correlation analysis

Descriptive statistics and correlation analyses were conducted using R, with the results presented in Table 11. As shown in Table 9, product familiarity was significantly and positively correlated with online shopping self-efficacy (*r* = 0.405, *p* < 0.001), emotional closeness to shopping-circle members (*r* = 0.453, *p* < 0.001), and willingness to disclose (*r* = 0.317, *p* < 0.001). Additionally, online shopping self-efficacy was significantly and positively correlated with willingness to disclose (*r* = 0.383, *p* < 0.001), as was emotional closeness to shopping-circle members (*r* = 0.358, *p* < 0.001).

**Table 11:** Correlation matrix for key constructs.

Variable	1	2	3	4	5
1 PF	—				
2 SCSC	0.489***	—			
3 SE	0.405***	0.337***	—		
4 EC	0.453***	0.349***	0.427***	—	
5 WD	0.317***	0.355***	0.383***	0.358***	—
Mean	3.8202	3.7719	3.7548	3.8260	3.7707
SD	0.97124	0.95662	1.01952	0.97922	0.96207

***p < 0.001, **p < 0.01, *p < 0.05; All reported correlations are Pearson coefficients.

## 6 Hypothesis testing

Consistent with the study’s single-occasion correlational design, all reported coefficients (e.g., “indirect effects,” “path coefficients”) represent statistical associations rather than causal effects. Terms such as “effect,” “impact, “and “influence” are used descriptively to refer to estimated statistical parameters, not to imply causal inference.

**Figure 2:**
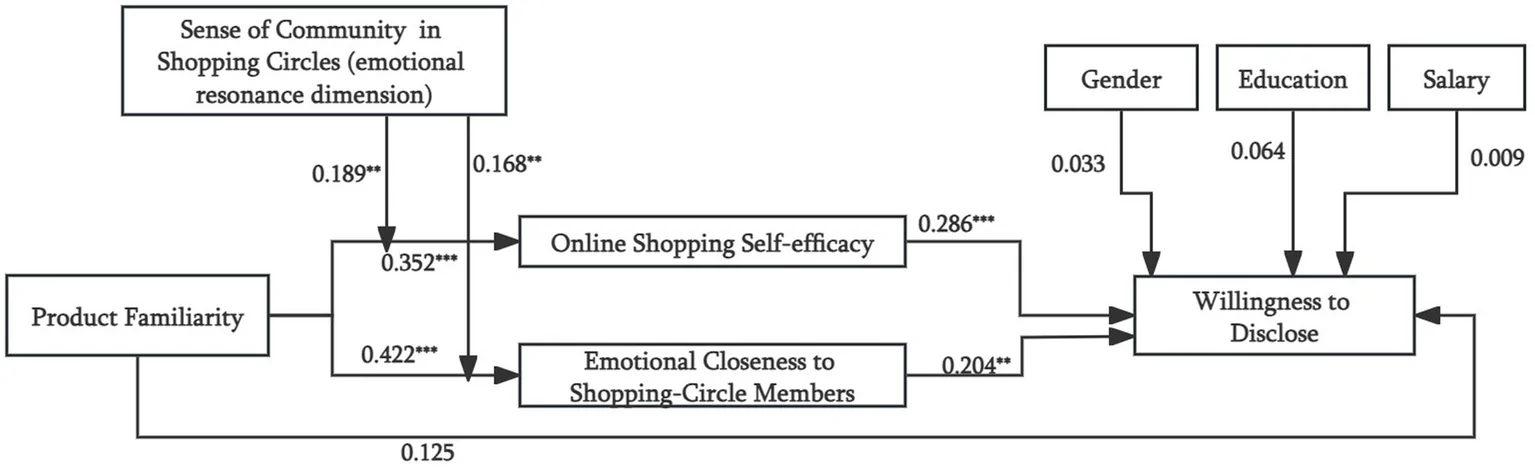
Results of path coefficient analysis. ** indicates *p* < 0.01, and *** indicates *p* < 0.001.

Path analysis is well-suited for examining the complex relationships among multiple variables, particularly in the presence of multiple mediating and moderating effects. Accordingly, this study employed covariance-based structural equation modeling (CB-SEM) for hypothesis testing. CB-SEM was selected because our study focuses on theory testing rather than prediction, and CB-SEM provides global model fit indices (e.g., CFI, RMSEA) that allow a comprehensive assessment of model adequacy (Reinartz et al., 2009). The results were calculated based on the parallel mediation model and the first-stage moderated effect test, as presented in Figure 2. Bias-corrected bootstrap (5,000 resamples) served as the primary inferential basis for testing moderated mediation (Hayes, 2015). Maximum likelihood estimation with robust standard errors (MLM) was used solely as a supplementary cross-validation check; all substantive conclusions are based on the bootstrap results. The results from both methods were consistent, providing additional descriptive support for the robustness of the findings.

### 6.1 Explanatory power of the structural model

Prior to testing the hypothesized paths, we assessed the explanatory power of the structural model. As presented in Table 12, the *R*^2^ values for online shopping self-efficacy (SE = 0.233), emotional closeness to shopping-circle members (EC = 0.284), and willingness to disclose (WD = 0.259) indicate moderate explanatory power. Following Cohen’s (1988) guidelines, the corresponding *f*^2^ effect sizes are 0.304, 0.397, and 0.349, reflecting medium to large effects. These results suggest that the model possesses satisfactory explanatory and predictive strength.

**Table 12:** Variance explained and effect sizes for endogenous variables.

Variable	*R* ^2^	*f* ^2^	Interpretation
SE	0.233	0.304	Medium
EC	0.284	0.397	Large
WD	0.259	0.349	Medium

### 6.2 Mediation effect test

Controlling for the effects of the control variables, product familiarity exerted a significant positive statistical association with both online shopping self-efficacy (*β* = 0.352, SE = 0.070, *p* < 0.001) and emotional closeness to shopping-circle members (*β* = 0.422, SE = 0.066, *p* < 0.001). Meanwhile, both online shopping self-efficacy (*β* = 0.286, SE = 0.066, *p* < 0.001) and emotional closeness to shopping-circle members (*β* = 0.204, SE = 0.071, *p* < 0.01) had a significant positive statistical association with willingness to disclose. To verify the mediating effects, the bootstrap method was employed to conduct path analysis on the dual mediation model using R. The results were cross-validated with the test outcomes derived from the maximum likelihood estimation with robust standard errors (MLM) estimator. All tests were based on the same parallel mediation model, and the unstandardized coefficients are reported herein, with detailed results presented in Table 13. The indirect effect of product familiarity on willingness to disclose through online shopping self-efficacy was 0.105, with a 95% confidence interval (CI) of [0.049, 0.176], which does not include zero. Additionally, the MLM estimation results indicated that the standard error of this effect was 0.031, with a *p*-value below 0.001. These findings confirm that the mediating effect is significant, thus supporting Hypothesis 1. Meanwhile, the indirect effect of product familiarity on willingness to disclose through emotional closeness to shopping-circle members was 0.089, with a 95% CI of [0.027, 0.158], which also excludes zero. The MLM estimation yielded a standard error of 0.032 for this effect, and the *p*-value was below 0.01. These results demonstrate that this mediating effect is also significant, thereby supporting Hypothesis 2.

**Table 13:** Results of bootstrap mediation effect test.

Variable	Effect value	95% CI lower bound	95% CI upper bound
Total effect	0.332	0.203	0.474
Total indirect effect	0.194	0.114	0.288
Decomposition of specific indirect effects			
PF → SE → WD	0.105	0.049	0.176
PF → EC → WD	0.089	0.027	0.158

### 6.3 Moderation effect test

**Figure 3:**
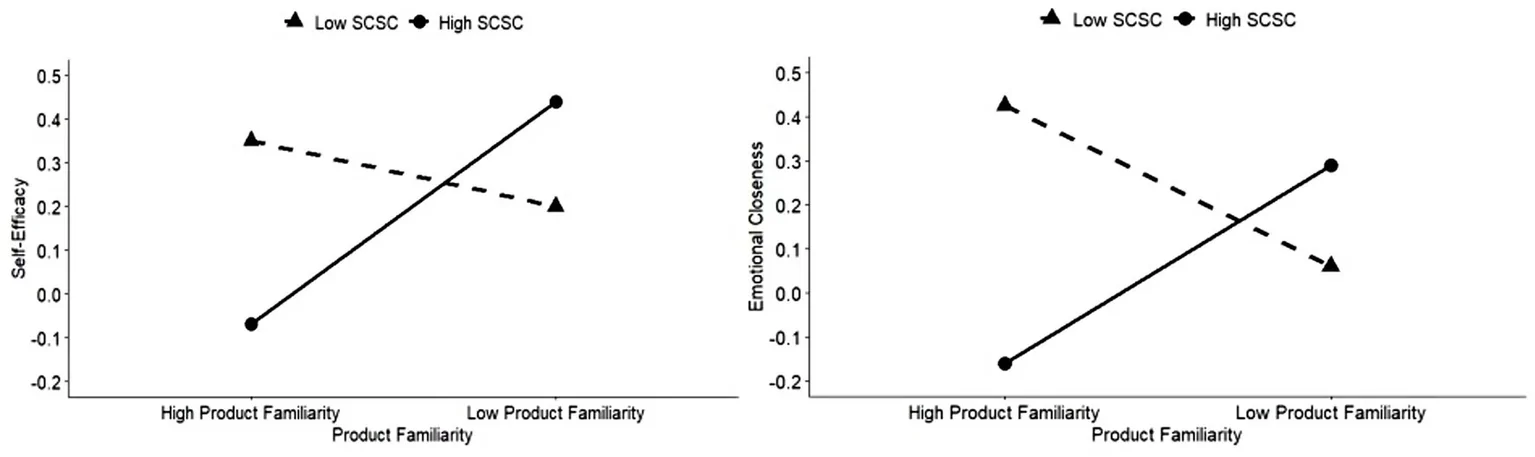
Moderating effects of circle community consciousness on the relationships between product familiarity and online shopping self-efficacy, and between product familiarity and emotional closeness to shopping-circle members.

As presented in Figure 3, the standardized interaction term between product familiarity and sense of community in shopping circles (emotional resonance dimension) showed a significant association with online shopping self-efficacy (*β* = −0.233, SE = 0.040, *p* < 0.001), indicating that sense of community in shopping circles (emotional resonance dimension) significantly moderates the relationship between product familiarity and online shopping self-efficacy. Similarly, the standardized interaction term between product familiarity and sense of community in shopping circles (emotional resonance dimension) had a significant effect on emotional closeness (*β* = −0.308, SE = 0.037, *p* < 0.001), demonstrating that sense of community in shopping circles (emotional resonance dimension) also significantly moderates the relationship between product familiarity and emotional closeness to shopping-circle members. To intuitively illustrate the moderating effects of sense of community in shopping circles on the relationships between product familiarity and online shopping self-efficacy, as well as between product familiarity and emotional closeness to shopping-circle members, moderating effect plots were constructed (see Figure 3). The results of the simple slope analysis revealed that when the level of sense of community in shopping circles (emotional resonance dimension) was high, the relationship between product familiarity and self-efficacy was not significant (*β* = 0.07, *p* = 0.33). In contrast, when the level of sense of community in shopping circles (emotional resonance dimension) was low, product familiarity was significantly and positively correlated with online shopping self-efficacy (*β* = 0.48, *p* < 0.001). These findings indicate that the moderating effect of sense of community in shopping circles (emotional resonance dimension) is negative, and thus, Hypothesis 3 is not supported. Furthermore, the simple slope analysis showed that when sense of community in shopping circles (emotional resonance dimension) was high, the relationship between product familiarity and emotional closeness to shopping-circle members was insignificant (*β* = 0.04, *p* = 0.52). Conversely, when sense of community in shopping circles (emotional resonance dimension) was low, product familiarity had a significant positive correlation with emotional closeness (*β* = 0.58, *p* < 0.001). This confirms that the moderating effect of sense of community in shopping circles (emotional resonance dimension) is negative, and therefore, Hypothesis 4 is supported.

### 6.4 Moderated mediation effects test

To test Hypotheses 5 and 6, the Bootstrap method was employed in this study. Specifically, we estimated the 95% confidence intervals (CIs) of the indirect effects of product familiarity on willingness to disclose self-information through online shopping self-efficacy and emotional closeness to shopping-circle members, respectively, under two different conditions (i.e., high vs. low levels of sense of community in shopping circles). Additionally, the 95% CIs of the differences in indirect effects between these two levels were also calculated. The detailed results are presented in Table 14.

**Table 14:** Results of moderated mediation effect analysis.

Mediator	Sense of community in shopping circles (emotional resonance dimension)	Indirect effect	95% CI lower bound	95% CI upper bound
Online self-efficacy	Low sense of community in shopping circles (emotional resonance dimension) (−1SD)	0.140	0.107	0.173
	High sense of community in shopping circles (emotional resonance dimension) (+1SD)	0.021	−0.031	0.077
	Difference	−0.119	−0.165	−0.070
Emotional closeness to shopping-circle members	Low sense of community in shopping circles (emotional resonance dimension) (−1SD)	0.115	0.097	0.133
	High sense of community in shopping circles (emotional resonance dimension) (+1SD)	0.009	−0.022	0.046
	Difference	−0.106	−0.136	−0.073

When the level of sense of community in shopping circles (emotional resonance dimension) was high, the indirect effect of product familiarity on willingness to disclose self-information through online shopping self-efficacy was 0.021, with a 95% CI of [−0.031, 0.077], which includes zero, indicating that the indirect effect was not significant. In contrast, when the level of sense of community in shopping circles (emotional resonance dimension) was low, the corresponding indirect effect was 0.140, with a 95% CI of [0.107, 0.173], which does not include zero, demonstrating a significant indirect effect. The difference in indirect effects between the high and low levels of sense of community in shopping circles (emotional resonance dimension) was −0.119, with a 95% CI of [−0.165, −0.070], which excludes zero. This result confirms that the difference in indirect effects was significant, indicating that the indirect effect under high sense of community in shopping circles (emotional resonance dimension) was significantly weaker than that under low sense of community in shopping circles (emotional resonance dimension). In other words, sense of community in shopping circles (emotional resonance dimension) exerts a negative moderating effect on this mediating path. Therefore, Hypothesis 5 is not supported. Similarly, when sense of community in shopping circles (emotional resonance dimension) was high, the indirect effect of product familiarity on willingness to disclose self-information through emotional closeness to shopping-circle members was 0.009, with a 95% CI of [−0.022, 0.046], which includes zero, suggesting no significant indirect effect. Conversely, when sense of community in shopping circles (emotional resonance dimension) was low, the indirect effect was 0.115, with a 95% CI of [0.097, 0.133], which does not include zero, indicating a significant indirect effect. The difference in indirect effects between the two levels was −0.106, with a 95% CI of [−0.136, −0.073], which excludes zero. This finding verifies that the difference in indirect effects was significant, reflecting a negative moderating effect. Thus, Hypothesis 6 is supported. Taken together, the differences in indirect effects across the two pathways constitute the indices of moderated mediation (IMM = −0.119, 95% CI [−0.165, −0.070]; IMM = −0.106, 95% CI [−0.136, −0.073]). Neither confidence interval encompasses zero, providing statistical evidence consistent with the view that sense of community in shopping circles (emotional resonance dimension) moderates both mediated pathways.

## 7 Discussion, implications, limitations and conclusion

### 7.1 Discussion

This study, grounded in social identity theory (Tajfel and Turner, 1986) and the theory of planned behavior (Ajzen, 1991), explores the relationship between product familiarity and Generation Z consumers’ willingness to disclose personal information. The results confirm that product familiarity is positively associated with willingness to disclose through the dual mediating paths of online shopping self-efficacy (H1) and emotional closeness to shopping-circle members (H2). This finding reveals that product familiarity is related to Generation Z consumers’ disclosure willingness through two distinct mechanisms. While self-efficacy represents a capability-based mechanism grounded in individuals’ positive evaluation of their information-processing capabilities (Alba and Hutchinson, 1987), emotional closeness to shopping-circle members constitutes an emotional bonding mechanism that reflects the emotional connection between Generation Z consumers and the product.

This study further investigates the boundary condition of sense of community in shopping circles (emotional resonance dimension). The results indicate that the positive relationship between product familiarity and emotional closeness to shopping-circle members is weaker when sense of community is higher, supporting H4. However, H3, which proposed a moderating role of sense of community on the relationship between product familiarity and online shopping self-efficacy, was not supported. The simple slope analysis revealed that the direction of the moderating effect was inconsistent with the hypothesis.

This non-significant result was not anticipated *a priori* and should be treated as exploratory. It can be tentatively interpreted in light of social identity theory (Ashforth and Mael, 1989; Tajfel and Turner, 1986). For individuals with a high sense of community in shopping circles (emotional resonance dimension), the strong situational cue of group norms may override the influence of product familiarity, directing cognitive focus toward collective values (Algesheimer et al., 2005; Dong et al., 2025). Consequently, the positive association between product familiarity and online shopping self-efficacy may be diminished, rendering online shopping self-efficacy a less effective mediating mechanism. In contrast, when sense of community in shopping circles (emotional resonance dimension) is low, the positive relationship between product familiarity and online shopping self-efficacy remains observable.

Similarly, the moderated mediation effect for the online shopping self-efficacy path (H5) was not supported, as the direction of the effect was inconsistent with the predicted hypothesis. This outcome underscores that the mediating role of online shopping self-efficacy is dependent on the positive association between product familiarity and online shopping self-efficacy. A high level of sense of community in shopping circles (emotional resonance dimension) reduces this association, and the finiteness of capability-based appraisal may further reduce attention to product familiarity (Petty and Cacioppo, 1986). These combined factors are associated with the invalidation of the indirect effect. In contrast, H6, which proposed that the indirect pathway via emotional closeness to shopping-circle members is weaker when sense of community (emotional resonance dimension) is higher, was supported. This pattern aligns with the theoretical reasoning that homogenization pressure and selective closure in high-community contexts may be associated with weaker emotional bonds (Baumeister and Leary, 1995).

### 7.2 Theoretical contributions

This study advances research on the mechanisms underlying the relationship between product familiarity and Generation Z consumers’ willingness to disclose personal information. Its core contribution lies in the empirical validation of a dual-mediation pattern. Existing studies in the field of consumer information disclosure often lack a systematic perspective on the relationships among variables (e.g., Cheung et al., 2015; Bélanger et al., 2021). Although product familiarity has been acknowledged as a key driver of consumer behavior (Johnson and Russo, 1984), the specific pathways through which it relates to disclosure willingness remain underexplored. Based on a dual-dimensional analytical framework, this study empirically confirms that product familiarity is positively associated with disclosure willingness through the parallel dual-mediation paths of online shopping self-efficacy and emotional closeness to shopping-circle members. This finding reveals the internal relationships of capability-based appraisal and emotional bonding mechanisms, thereby providing a more systematic multi-path mechanism analysis paradigm for subsequent related research.

Another significant contribution lies in the investigation of the boundary conditions of sense of community in shopping circles (emotional resonance dimension) within the domain of Generation Z consumer behavior, clarifying its differentiated moderating effects across the dual mediating paths. Previous studies exploring the effects of product familiarity have largely overlooked the heterogeneous moderating role of circle characteristics (Jin et al., 2018; Dong et al., 2025). While research on virtual communities has established the importance of sense of community (emotional resonance dimension) in shaping user behavior (Blanchard and Markus, 2004; Koh and Kim, 2003), its application as a moderator in the context of product familiarity and information disclosure remains limited. This study reveals that sense of community in shopping circles (emotional resonance dimension) exerts a negative moderating effect on the relationship between product familiarity and emotional closeness to shopping-circle members (supporting H4 and H6), but not on the relationship between product familiarity and online shopping self-efficacy (H3 and H5 not supported). These findings verify the differentiated boundary roles of sense of community in shopping circles (emotional resonance dimension) within the dual-mediation process, thereby extending the theoretical application of circle characteristics in consumer behavior research.

In addition, this study refines the theoretical understanding of the differentiated moderating logic across the dual mediating paths in the context of information disclosure. While existing research has examined combined mediation and moderation mechanisms, with some attention to moderating differences across dual paths, such discussions have largely centered on traditional variables such as perceived benefits and perceived risks (Cheung et al., 2015; Acquisti et al., 2015; McKee et al., 2024). In contrast, there remains a notable gap in the analysis of moderating effects involving typical social context variables of Generation Z, such as sense of community in shopping circles (emotional resonance dimension). Through empirical testing, this study distinguishes the differentiated moderating effects of sense of community in shopping circles on the two mediating paths: the emotional closeness to shopping-circle members path is negatively moderated, whereas the online shopping self-efficacy path shows no significant moderating effect. This finding not only enhances the theoretical precision of the formation mechanism of disclosure willingness but also provides a new basis for integrating dual-mediation and moderation models in consumer behavior research.

Notably, recent research (Dai et al., 2026) indicates that consumers prefer human customer service agents when disclosing subjective information (e.g., preferences, feelings), whereas they prefer AI agents when disclosing objective information (e.g., height, weight). This finding suggests that the dual pathways associated with product familiarity may vary across different types of information. The present study finds that product familiarity is associated with Generation Z’s willingness to disclose through online shopping self-efficacy and emotional closeness to shopping-circle members. Integrating these two lines of inquiry, future research could further explore whether product familiarity and information type are jointly associated with Generation Z’s disclosure behavior across different shopping circles.

### 7.3 Managerial implications

This study centers on the relationship between product familiarity and Generation Z‘s willingness to disclose personal information, with a particular focus on the mediating roles of online shopping self-efficacy and emotional closeness to shopping-circle members, as well as the moderating role of sense of community in shopping circles (emotional resonance dimension). The findings reveal that product familiarity is positively associated with disclosure willingness through two distinct mechanisms. Specifically, online shopping self-efficacy serves as the capability-based pathway, while emotional closeness to shopping-circle members functions as the emotional bonding pathway. Moreover, sense of community in shopping circles is associated with a weaker emotional closeness pathway but does not significantly moderate the capability-based pathway (online shopping self-efficacy), highlighting the contextual sensitivity and differentiated nature of the proposed mechanisms. Building on these insights, several practical implications for product management emerge.

A key implication for product managers lies in leveraging the dual pathways of product familiarity by constructing a user connection system that integrates capability-based and affective dimensions. Given that product familiarity is associated with disclosure willingness through online shopping self-efficacy and emotional closeness to shopping-circle members, brands should reinforce product familiarity from both angles. From a capability-based perspective, brands can facilitate user understanding through scenario-based information delivery, enabling users to fully grasp product information and thereby supporting their positive self-assessment of information-processing capabilities (Bartol et al., 2023; Zhang C. et al., 2021; Zhang Y. et al., 2021). From an affective perspective, brands should enhance interactive experiences to foster emotional bonding (Epstein et al., 2025; Baumeister and Leary, 1995), leveraging high-frequency interactions to strengthen users’ emotional identification with their shopping circles (Wenzel and Benkenstein, 2019). In addition, brands need to adapt their product information dissemination formats to align with users’ receptivity habits, thereby supporting the positive association of product familiarity (Heo and Lee, 2025; Hu and Ou, 2025).

A related implication stems from the heterogeneous nature of sense of community in shopping circles (emotional resonance dimension), which calls for multi-level user operation strategies. The results indicate that sense of community in shopping circles is associated with a weaker relationship between product familiarity and emotional closeness to shopping-circle members, but does not significantly moderate the relationship between product familiarity and online shopping self-efficacy. Accordingly, brands should tailor their operational approaches to Generation Z users with varying levels of circle consciousness. For users with a high sense of community, brands should be aware that emotional bonding pathways may be less strongly associated with disclosure willingness due to group norms and homogenization pressure; thus, they may need to leverage alternative mechanisms such as collective identity rather than relying solely on emotional closeness. For those with a low sense of community, brands can focus on building emotional bonds directly, as this pathway remains observably associated (Mason et al., 2022). Additionally, brands should establish a dynamic identification mechanism for circle characteristics and adjust their operational strategies in real time to accommodate shifts in users’ circle consciousness (Su et al., 2025; Tseng et al., 2025).

The findings further suggest that product managers can more effectively support users’ information disclosure behaviors by attending to the distinct regularities of the dual mediating paths. This study clarifies that the online shopping self-efficacy path is consistently associated with disclosure willingness across different levels of sense of community (emotional resonance dimension), whereas the emotional closeness to shopping-circle members path is more context-dependent. For users who are more readily oriented toward self-efficacy, brands can design activities that allow users to verify their own usage capabilities, thereby supporting their usage confidence through functional benefits and potentially increasing their willingness to proactively disclose information (Bartol et al., 2023). For users who place greater emphasis on emotional closeness, brands should be mindful that this pathway may be less strongly associated with disclosure willingness in high-community contexts; thus, alternative strategies may be needed. In addition, brands should strengthen their information security assurance mechanisms (Bélanger et al., 2021) to address users’ concerns about information disclosure, thereby providing trust-based support for both mediating pathways.

### 7.4 Limitations and future research

Although this study offers clear contributions, several limitations should be acknowledged to inform future research directions.

First, the sample was drawn from a single online panel (Wenjuanxing) and restricted to respondents aged 18 and over, which was implemented through a platform-level pre-screening filter that allowed only individuals born between 1997 and 2009 to participate, thereby excluding younger Generation Z consumers (born after 2012). Consequently, exact numerical age statistics (i.e., mean and standard deviation) are not available in the dataset, as age was used solely as a screening criterion rather than collected as a separate questionnaire item. In addition, the sample was highly educated (approximately 95% held a bachelor’s degree or higher), which is not representative of the broader Generation Z population. These demographic skews, together with self-selection into an online panel, limit the generalizability of our findings. Future research should replicate our results using more diverse samples. to enhance external validity. In particular, the absence of continuous age data (mean, SD) due to the pre-screening filter prevents more fine-grained age-related analysis; future research could collect age as a continuous variable to enable such examinations.

Second, although we provided empirical support for combining the two subsamples via MG-CFA, our scale development procedure did not use a fully independent sample for confirmatory factor analysis. Future research could adopt a stricter two-sample design with separate EFA and CFA subsamples to further validate the factor structure.

Third, the measurement approach also has limitations. The constructs of sense of community in shopping circles and emotional closeness to shopping-circle members were assessed via self-reported questionnaires, a method that may be subject to individual cognitive biases. Although our post-hoc tests (Harman‘s single-factor test and CLF analysis) suggested that common method bias was not a serious concern, the self-report nature of the data remains a limitation. To improve measurement accuracy, future studies could combine survey data with behavioral tracking and consider multi-source designs to address these issues. Moreover, our findings should be interpreted within the specific operationalization of the constructs (e.g., the emotional resonance dimension of sense of community), and caution should be exercised when generalizing to the broader theoretical definitions (e.g., belonging, identification, influence, and membership).

Fourth, the cross-sectional design adopted here does not allow for the identification of causal relationships over time. This is an important caveat: the findings should be interpreted as demonstrating statistical associations rather than causal effects. Furthermore, this design cannot rule out reverse causality or reciprocal relationships. For example, willingness to disclose may also increase product familiarity through post-purchase learning and feedback loops, rather than product familiarity unidirectionally predicting disclosure.

Fifth, this study did not model several plausible confounders that may account for shared variance among the focal variables, including privacy concern, digital literacy, and product trust. These constructs could influence both product familiarity and willingness to disclose, and their omission represents a limitation of the current analysis.

To address these limitations, longitudinal research would be helpful to track changes in the key variables and better establish temporal order. Moreover, future work could incorporate other relevant factors—such as digital literacy or product trust—and examine how they relate to sense of community in shopping circles to further extend our understanding of how product familiarity is associated with willingness to disclose, as well as collect continuous age data for more fine-grained analysis.

## 8 Conclusion

This study, grounded in social identity theory and the theory of planned behavior, examined how product familiarity is associated with Generation Z consumers’ willingness to disclose personal information, with online shopping self-efficacy and emotional closeness to shopping circle members as dual mediators and sense of community in shopping circles (emotional resonance dimension) as a moderator. The results indicate that product familiarity is positively associated with disclosure willingness through the parallel mediating paths of online shopping self-efficacy and emotional closeness, confirming both capability-based and emotional bonding mechanisms. Furthermore, sense of community in shopping circles (emotional resonance dimension) is associated with a weaker emotional closeness to shopping-circle members path (supporting H4 and H6), but does not significantly moderate the online shopping self-efficacy path (H3 and H5 not supported). By clarifying these parallel mechanisms and their differentiated boundary conditions, this study contributes to a more nuanced understanding of consumer information disclosure in the context of social identity and group dynamics, offering both theoretical insights and practical guidance for brands seeking to foster user engagement.

## Data Availability

The raw data supporting the conclusions of this article will be made available by the authors, without undue reservation.

## Footnotes

1
https://www.wjx.cn/
