# Correction: Online shopping self-efficacy and emotional closeness to shopping-circle members as dual pathways: the association between product familiarity and Generation Z consumers' willingness to disclose

**DOI:** 10.3389/fpsyg.2026.2007638

**Published:** 2026-10-08

**Authors:** Mi Shi, Lanxi Wang, Miao Guo, Mengxian Chen

**Affiliations:** School of Economics and Management, Tiangong University, Tianjin, China

**Keywords:** emotional closeness to shopping-circle members, online shopping self-efficacy, product familiarity, sense of community in shopping circles, willingness to disclose

There was a mistake in Figure 2 as published. The control variable “Gender” appeared twice, the second occurrence should read “Education”. The corrected Figure 2 appears below.

**Figure 2:**
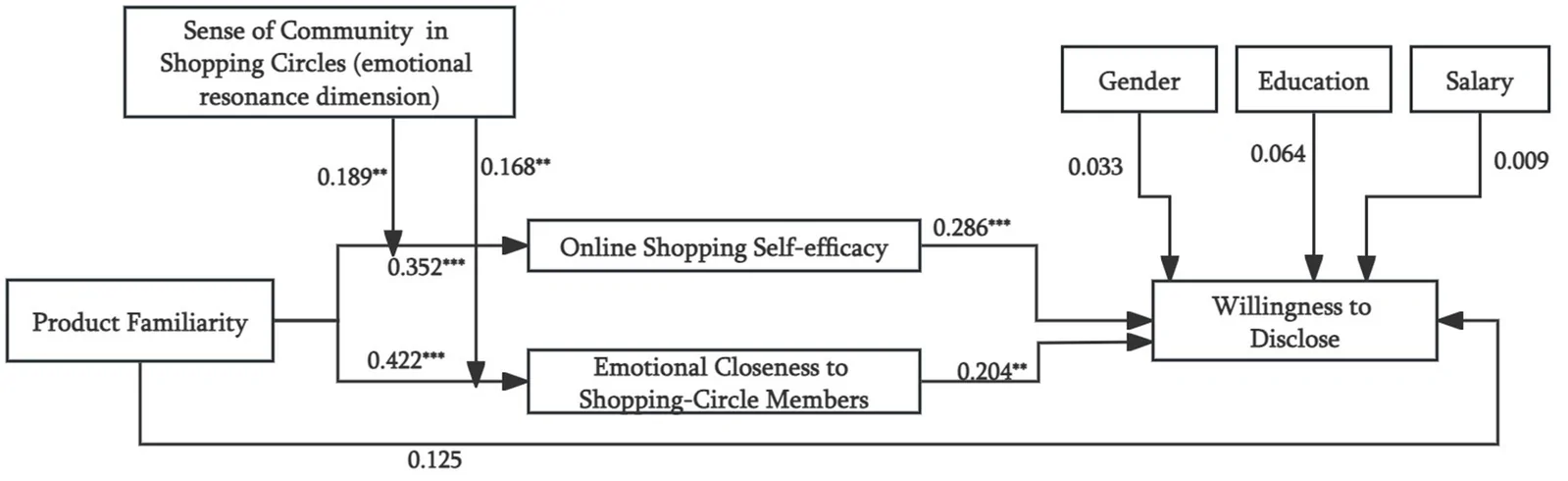
Results of path coefficient analysis. ** Indicates *p* < 0.01, and *** indicates *p* < 0.001.

The original version of this article has been updated.

