# Supplementary material for: Online shopping self-efficacy and emotional closeness to shopping-circle members as dual pathways: the association between product familiarity and Generation Z consumers’ willingness to disclose
Source: Front Psychol. 2026 Sep 9;17:1851651. doi: 10.3389/fpsyg.2026.1851651 (PMC13597866; doi:10.3389/fpsyg.2026.1851651)
Supplement: Supplementary file 1 [file Supplementary_File_1.pdf]

Appendix A

The measurement items of constructs

**Main constructs and measurement items**		**Sources**
Product Familarity (PF)		Adapted from Loureiro et al.(2020)
PF1	I am very familiar with the products recommended in my shopping circle.	
PF2	I can accurately identify the differences between products discussed in my shopping circle.	
PF3	I have had multiple experiences using products recommended by my shopping circle.	
PF4	I am familiar with how to participate in group buying or shared reviews in my shopping circle.	
PF5	I have a good understanding of the product quality standards within my shopping circle.	
Emotional Closeness to Shopping-Circle Members (EC)		Adapted from Kong and Liang. (2017)
EC1	Purchasing products through my shopping circle makes me feel close to its members.	
EC2	Product recommendations from my shopping circle members are an important part of my consumption life.	
EC3	I identify with the product reviews shared by members of my shopping circle.	
EC4	I feel a sense of closeness with shopping circle members regarding our consumption preferences.	
EC5	Sharing focus on the same products brings me emotional closeness with my shopping circle.	
Online Shopping Self-efficacy (SE)		Adapted from Liu et

Main constructs and measurement items		Sources
SE1	I can always solve tricky problems encountered in shopping circle transactions if I put in the effort.	al.(2017)
SE2	When facing difficulties in shopping circle interactions, I can come up with solutions.	
SE3	I have the ability to handle unexpected incidents during shopping circle purchases.	
SE4	I can find hard-to-get items through various channels within my shopping circles if I want to.	
Sense of Community in Shopping Circles (emotional resonance dimension)		Self-developed based on McMillan and Chavis (1986); Jin et al. (2018)
SCSC1	I find that I share similar emotional responses with members of my online shopping circle.	
SCSC2	In general, my emotional expressions align with those of other members in my shopping circle.	
SCSC3	When facing shopping situations, my emotional reactions are consistent with my shopping circle members.	
SCSC4	I feel that I share similar emotional experiences and behavioral responses with my shopping circle members.	
SCSC5	Typically, my emotional feedback patterns in shopping contexts are similar to those of my shopping circle members.	
SCSC6	There is a strong emotional resonance between me and my shopping circle members.	
Willingness to Disclose		Adapted from Malhotra et al. (2004)
WD1	I intend to use my real identity when joining shopping circles for products I have purchased.	

Main constructs and measurement items		Sources
WD2	I would be willing to proactively provide my real personal information to my shopping circle members.	
WD3	I am comfortable with interacting with other shopping circle members without hiding my real identity.	
WD4	It is acceptable for me to post reviews using my real information in my shopping circles.	
WD5	I plan to share my real consumption preferences (e.g., product choices, purchase history) with my shopping circle.	
WD6	I don't mind providing my real personal information in surveys initiated by my shopping circle.	
